# Intergenerational narratives of toxicity: understanding heterogeneity and care in a polluted steeltown (Taranto, Italy)

**DOI:** 10.1057/s41292-025-00364-3

**Published:** 2025-08-25

**Authors:** Raffaele Ippolito, Carmen Sale, Maaret Jokela-Pansini

**Affiliations:** 1 School of Geography and the Environment, University of Oxford, S Parks Rd, Oxford, OX1 3QY UK; 2 LUMSA, Rome, Italy

**Keywords:** Intergenerational narratives, Toxicity, Pollution, Care, Taranto

## Abstract

This article examines environmental narratives amidst chronic industrial pollution across three generations in Taranto, Italy. Drawing on ethnographic research with residents positioned in different historical periods, we show how each generation’s understanding of toxicity is intimately tied to shifting economic conditions, political interventions, and embodied experiences in Taranto’s polluted landscape. The first generation, closely tied to state-led industrial development, recalls their experience of pride and modernity. The second generation is faced with growing scientific evidence on industrial pollution and institutional scrutiny: they make sense of toxicity by questioning the promise of prosperity that the industrial development and resulting modernity offered. The youngest generation, who grew amid persistent environmental degradation, creates a narrative of pollution as a given dimension of everyday life and one that calls forth community cohesion. By highlighting these intergenerational narratives and their ongoing renegotiations, we shed light on how wellbeing and care are assembled, reworked, and contested over time. In doing so, this paper contributes to more heterogeneous understandings of environmental justice and the ways communities live through, and make sense of, industrial harm.

## Introduction

“See? It’s everywhere—we are not so close, but it [the dust] gets here as well!” said Giovanni while showing the black dust he collected on his finger from the rooftop of his apartment building. The dust had shiny particles, namely iron dioxide. We were just a few kilometers away from the factory. Giovanni said it was risky to touch the dust, but it didn’t matter, because when he worked in the factory his whole body used to get covered in it, he even inhaled it. “They gave us masks, but nobody really used them.”

Raffaele (co-author) met Giovanni through a friend of his, Massimo, who was Giovanni’s nephew. Later that day, Raffaele told Massimo that he had met his uncle and that he showed him around his house. When he mentioned to Massimo that he was interested in understanding how different generations understood and related to pollution in different ways, Massimo paused for a few seconds. “You know, it’s not simply because they were exposed to pollution all the time, it’s also that they were making very good money working there. It’s not the same for us—now we are aware of the effects of pollution, but the jobs at the factory are also fewer. There is nothing else here, and it’s because of the wealth that the factory used to produce.” Raffaele and Massimo chatted about this for the next hour, reflecting on how residents from different generations were so intimately connected and deeply cared about each other, yet their narratives of industrial pollution could not be further apart. Massimo expressed concern about having a child one day: “I’d be worried about raising them here, it’s a mess.”

When it comes to the steelworks of Taranto, one of the largest in Europe, residents of the neighboring areas have different opinions, as the tension between Giovanni and Massimo illustrates. To some of them, pollution is the ultimate symptom of environmental injustice, one in which the Italian state is complicit. Environmental groups claim that environmental injustice has become undeniable under the United Nation’s designation of Taranto as a “sacrifice zone” (UN OHCHR 2022) and the verdict of the European Court of Human Rights (ECHR) fining the Italian government for violating the rights of Taranto’s residents (Greco 2020). To other residents, however, industrial pollution and its adverse health effects on the local population (Pirastu et al. 2013) is a price to pay for the industrial and economic development in the city, which, up until the 1960s, was one of Italy's poorest areas (Barca and Leonardi 2018). What these different positions share is the acknowledgment that the city’s history and landscape are inherently linked to the factory (see also Romeo 2019, for an extensive historical analysis).

After 3 years of individual ethnographic research in Taranto , our work has documented the complex and diverse experiences of toxicity within the city. Our individual enquiries broadly focused on how different groups of people in Taranto experienced and acted on industrial pollution, with specific foci on justice (Ippolito 2022), reproductive health (Jokela-Pansini 2022) and children’s agency (Sale 2024). Through a research group on Taranto we set up in 2023, we found similar thematic overlaps between our individual projects and became interested in comparing how residents from different neighborhoods and age groups acted on industrial pollution in the city. One year later, some of our collective work has addressed the heterogeneity of Taranto’s environmental narratives across different neighborhoods of the city (Jokela-Pansini et al. 2024).

In this paper, we move our analysis of toxicity in Taranto away from the neighborhood-level and shift our focus to another analytical axis: time. By doing so, we build on anthropological and geographical research on time–space relationships (Massey 2005; May and Thrift 2003), which highlight temporality as a key dimension for understanding the connectedness between humans and their (toxic) environment (Davies 2019; Nixon 2011) and the impact of time on agency and political action (Ho 2021). When we consider industrial pollution in relation to the everyday experiences and actions of affected communities, then, the ‘when’ matters as much as the ‘where’ (Ghertner 2017, see also Ho 2021).

The aim of our paper is to understand how residents across different generations relate to the industrial landscape of Taranto based on their witnessing of different phases of industrial development, and how these experiences are shaped by the landscape in a dynamic relationship. With landscape, thus, we do not merely mean a static background for human interaction but its reflexive relationship with the social processes within which it is embedded (Mitchell 2002). With the term ‘generation,’ we refer to residents who share a lived experience of a specific environment at a specific time. Generational experiences are situated in specific socio-political and economic contexts and broad periods of Taranto’s industrial history spanning from 1960 to 2022. We use the analytical category of generation because participants—regardless of their background—often described their experiences as both shared and specific to their own generation, contrasting them with the experiences of younger and older cohorts.

Inspired by scholarship on passing toxicity as ‘embodied harm’ (Mitchell 2002), including Lamoureaux’s work on “(inter)generational toxicology” (2021), we move away from the epigenetic analysis to imagine toxic relationalities and “public perceptions of harm at scales that exceed the individual” (540). Rather than focusing on the biological impact of toxins and their biosocial impact on future generations (see for example Gibbon and Lamoreaux 2021; Lappé and Jeffries Hein 2021), we are interested in understanding how toxicity may be examined as a narrative produced and reproduced in an intergenerational dialogue.

Here, we build on Newman’s (2012) idea of ‘toxic autobiography’ to bring attention to toxicity not as abstract, but rather as “highly personal and communal, accounted for in stories of hazardous geographies, biographies, and histories” (42). Toxic autobiography, Newman argues, flows from a deep sense of “crisis among marginalized groups of people […] that feel trapped in landscapes well beyond mainstream concern” (22). Although Newman’s study of toxic autobiography is a literary project aimed at advancing environmental justice, we take inspiration from his approach to document the ‘unwritten’ stories of generations of residents that are often underrepresented in environmental debates (Mah 2016; Vasudevan 2012). These stories, we argue, are written on the bodies and places that residents daily experience and reproduce.

Methodologically, our paper combines different approaches and positionalities. Two of us—Raffaele and Carmen— are from Taranto and were born and raised in the city, while Maaret has been connected to the Apulian region through family ties for 20 years and spent over 5 years living in the region. We draw on ethnographic research conducted between 2019 and 2023. Maaret and Raffaele have collected data through participatory and art-based methods, interviews and participant observation with over 80 members of civil society groups and residents. To this Carmen has integrated a participatory storytelling exercise conducted with 120 children in Tamburi, Taranto’s district closest to the steelworks. Our individual research protocols have been reviewed and approved by ethics committees in Italy (LUMSA CERS, Approval Reference 8/2022) and the UK (University of Oxford CUREC, Approval References SOGE-2020-1A-38 and SOGE-2020-1A-213).

The empirical sections of this paper retrace generational narratives across three stages of Taranto’s industrial development. To each of these ‘official’ historical accounts of Taranto’s development, found also in previous literature (Banini and Palagiano 2019; Barca and Leonardi 2016; Greco and Di Fabbio 2014; Romeo 2019), we connect stories that we found central to defining the shared generational experiences. We broadly divide the three generations as: (1) the generation that witnessed the birth of the steelworks as a state-led project of national development (1940–1970), (2) their children’s generation, who experienced both wealth and the emergence of a public health crisis in the city (1971–2000), and (3) their grandchildren’s generation, who are growing up in a condition of late-industrialism (2001–present) (see Fortun 2012).

We argue that these heterogeneous narratives shed light on the importance of understanding pollution and toxicity more broadly as an intergenerational experience of economic, political, and social transformation. Though acknowledging that using ‘generation’ as an analytical category can be varied and sometimes inconsistent (Vanderbeck, 2007), we mobilize the notion to temporally position Taranto’s people in relation to one another, emphasizing their specific time-contingent attachment to the history of industrial development of the city. Rather than essentializing experiences of pollution across generations, this paper thus seeks to offer a critical outlook on studies of toxicity and environmental justice. It does so by illustrating that the residents’ contrasting attachments, which can coexist within harmfully polluted landscapes, do not divide communities, but on the contrary, unfold as a project of intergenerational care.

With chemical harm being often at the center of scholarship on environmental injustice (Liboiron et al. 2018; Murphy 2019; Nading 2020), our paper builds on previous work focusing also on the positive attachments, which are created and sustained via industrial contamination (see also, for example, Tironi and Rodríguez-Giralt 2017). Taken in relation to one another, the intergenerational heterogeneity (and seeming fragmentation) of environmental narratives studied in Taranto emerges as a coherent narrative that reconciles both the chemical harm and the care enabled by industrial development.

## Constructing temporalities and narratives of toxicity

At the individual and community levels, industrial toxicity is often characterized by a temporal disconnect between exposure and the onset of illness. For example, in Taranto, industrial activity began in the 1960s, but it was only in the early 2010s that the first epidemiological studies started highlighting a significant correlation between pollutants and health issues in the local population (Mangia et al. 2013; Pirastu et al. 2013). While these studies prompted the rise of environmental advocacy, this temporal disconnect—both experiential and clinical—creates dissonance in the perception of industrial harm, in some cases making communities resigned to living with it (Auyero and Swistun 2009; Ippolito 2022; Lora-Wainwright 2021).

If on the one hand, this ‘slow violence’ can be invisible to the outsider’s eye (Nixon 2011), communities living with it have developed ways to cope and/or resist that do not fit conventional ideas of activism (Davies 2019; Lora-Wainwright 2021). For example, Thom Davies’ (ibid.) notion of ‘slow observation’ recognizes that a temporally diluted experience of chemicals foregrounds an alternate epistemology to understand and counter environmental injustice.

Despite being aware of their toxic surroundings, some communities frequently feel unable to enact change but still engage in everyday resistance. Some studies have found that individuals may choose to exclude pollution from their environmental narratives to reinforce their sense of wellbeing despite toxicants (Jokela-Pansini et al. 2024; Lou 2022). Other research has emphasized the small, personal actions that people undertake to mitigate environmental degradation in their everyday lives (Davies 2019; Shapiro 2015; Valdivia 2018). Anna Lora-Wainwright’s (2021) concept of ‘resigned activism’ refers to individualized or family-oriented tactics aimed at minimizing pollution in one’s immediate surroundings by buying bottled water, keeping windows shut, wearing masks, and turning down risky jobs. “Resigned activism is not a starting point; instead, it is learned state of being. These individualized and family-oriented strategies should not be written off as selfish, as they are often a last resort” (ibid). Speaking from the context of Chile’s most contaminated industrial zone, Lora-Wainwright’s (2021) idea is echoed by Tironi (2018), who shows how residents of Los Maitenes contest industrial toxicants, though without engaging in direct resistance. Here the idea of ‘intimate activism’ describes a response to toxicity that is “incremental, chronic and inseparable from life itself” (451) and “lived in the rhythm of ordinary corrosion and decay, in the nondescript temporality of chronicity and continuity” (442).

Taken together, this scholarship calls for an expansion of our understanding of agency in the face of environmental harm, shifting attention to everyday perceptions and attitudes that, while arguably unspectacular, are underpinned by the multifaceted complexity of local realities. Our paper contributes to advancing this debate by stressing that understanding ‘resigned’ and ‘intimate’ agency in polluted communities requires stretching the temporal scale of analysis across different generations. Through this lens, we argue, we can move away from an understanding of heterogeneous narratives of toxicity as a symptom of community and moral fragmentation. Instead, our analysis offers an understanding of the diverse attitudes and positions on industrial pollution as a product of an intergenerational dialogue shaped by care.

Below, we build on this extensive research viewing the temporalities and practices that residents daily negotiate to live in industrially polluted areas and seek to understand how different temporal experiences of the industrial area have produced different narratives of toxicity across the three generations.

### 1st generation (1940–1970)—‘everybody liked the golden goose’: industrialization and wealth

Following the 1946 referendum that transitioned Italy from a monarchy to a republic, the newly formed Italian government implemented policies to drive industrialization, particularly focusing on the underdeveloped South (Zamagni 1993). In 1950, the establishment of the Fund for the South (Cassa per il Mezzogiorno) was a critical step in this direction, designed to modernize the southern regions through rapid industrialization (Ammanati 1981). Up until this point, Southern Italy, including the Apulian region where Taranto is located, was largely agrarian, which contributed to a significant economic disparity between the industrialized North and the agricultural South (Zamagni 1993). The Italian steel industry experienced substantial growth during the 1950s and prompted the government to build a fourth steel mill to enhance national production capacity and produce “steel in the place of the olive trees” (Romeo 2019). Taranto was selected as the site for this new steelworks due to its strategic port location and the economic downturn it faced post-World War II, with diminished demand for maritime warfare activities (Pignatelli 1976). The state-owned enterprise Italsider spearheaded the establishment of the steel plant in the early 1960s. By 1964, the steel plant was operational and significantly boosted local employment and economic activity. Additional investments followed, including the development of a petrochemical refinery adjacent to the steelworks (Pierri 1997).

Together with the factory, the city also transformed. Taranto extends over 15 km along the coast of the Gulf of Taranto. The new industrial facilities were established in the Northern part of the city over a land extension that was nearly three times as large as the city itself. The Tamburi and Paolo VI neighborhoods, two districts also in the Northern part of the city, saw the biggest transformation in response to industrialization. Large housing projects completely transformed the two neighborhoods, offering accommodation to workers relocating both within the city but also from neighboring towns and other areas of Southern Italy. The expansion of industrial buildings and workers’ housing over the urban landscape set the scene for the uneven distribution of environmental burden within the city, with the working class bearing the greater burnt of chemical exposure, and areas in the Southern part of the city being more moderately affected.

**Fig. 1:**
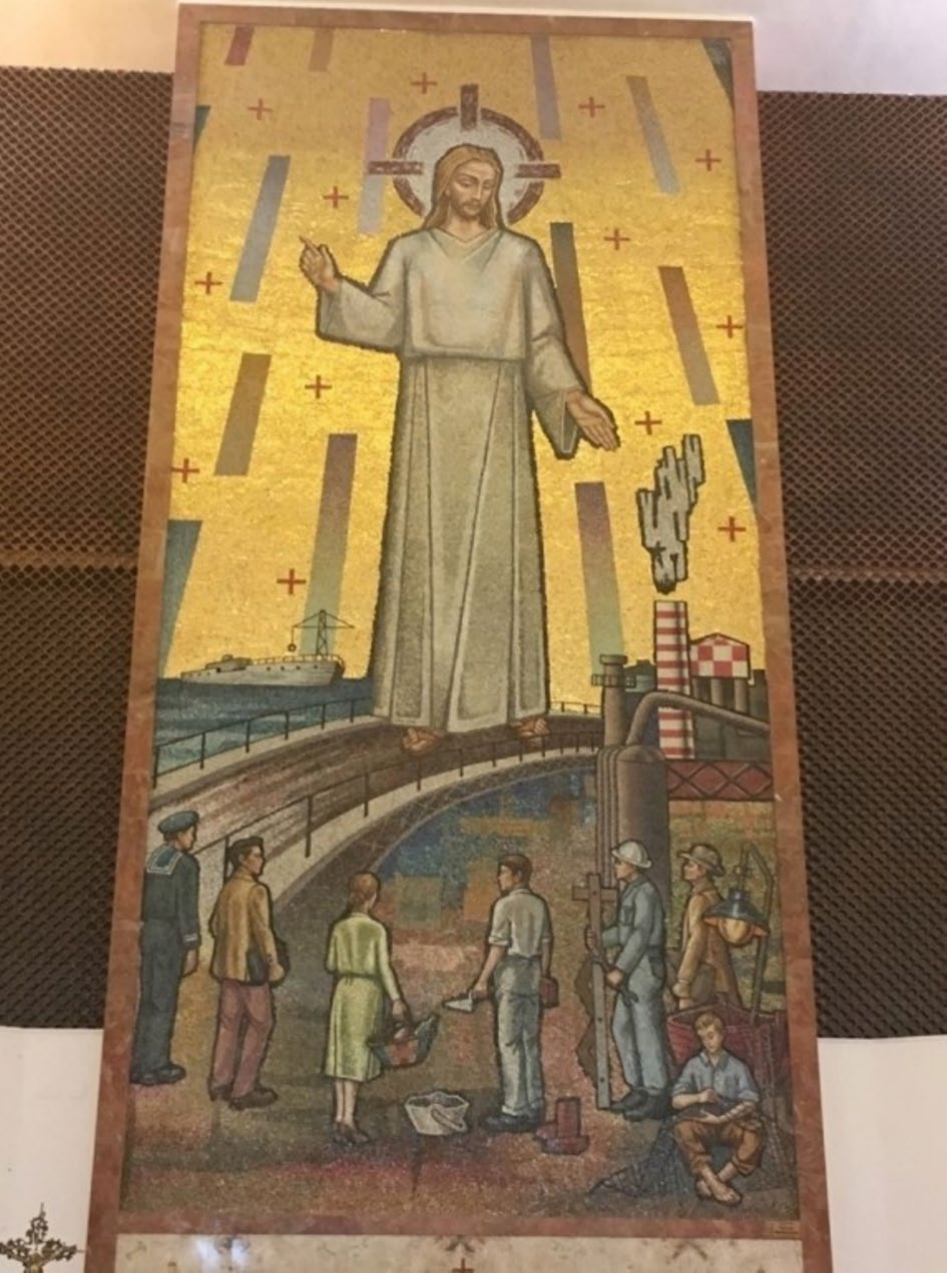
Jesus Heavenly Worker. Photo by Raffaele Ippolito

Throughout the 1960s and 1970s, the steel plant rapidly expanded and became one of Europe's largest integrated steelworks. Its capacity grew from two million tons of unrefined steel in the mid-1960s to 4.5 million tons per year by the early 1970s (Ammanati 1981). This industrial boom brought substantial employment opportunities, with around 25,000 people directly employed by the factory and another 14,000 working in related industries. The steelworks fundamentally reshaped Taranto’s economy and identity and transformed it from a primarily agricultural society into an industrial powerhouse. The mosaic in the Church of Gesù Divin Lavoratore (Jesus Heavenly Worker)—a few hundred meters away from the factory’s smokestacks—epitomizes this transformation, depicting Jesus among modern professionals, symbolizing the new industrial identity bestowed upon the community by the steel plant (Fig. 1—see also (Ippolito and Walther 2019).

The generation that experienced this period of Taranto’s industrial development is largely made up of people in their 60s, 70s and 80s at the time of data collection. Compared to the other two generations, these participants were more likely to have experienced both the poverty of the aftermath of WWII and the boom of the Italian economy in the 1970s and 1980s, which in their case materialized through the process of industrialization.

Giuseppe, a retired factory worker, remembered with a smile the period of State-ownership of the factory. He told us that those were years of hard work and joy, given that he had a very well-paid job, “with a salary four times higher than that of a high school teacher!” In recalling his satisfaction with his job, he summarized the working conditions in the factory during the period of state ownership (19961–1995):

[...] when the State was managing the factory, everybody liked the golden goose. There was so much money, so much money! ILVA used to be a money factory. It used to produce so much...

Much like Giuseppe, other former workers interviewed expressed great content with their salaries during the public phase of Ilva, recalling a sense of pride associated with their status as workers. We observed that industrialization in Taranto, in addition to increasing the overall wealth of the city and many of its people, enabled the formation of a modern identity among the earlier generations of workers and their families. Another resident explained:

The skilled workforce of Taranto was very well known in the Ilva’s industrial parks of Genova and Bagnoli, because there was some precious knowledge that was passed on. Tarantini workers were proud of this. Working the steel, working it in a certain way, shaped the identity of the community. Today that mechanism of identity creation is lacking, and new generations care less about the question of identity. To them, it’s more about making money to own certain things thanks to the factory, and on the other hand they care more about pollution.

Within this phase of industrial development in Taranto, we observed how workers were well respected because of this higher economic and professional status. For example, some participants mentioned that it was common for experienced workers to travel abroad to supervise wielding for major construction projects, highlighting the importance of Taranto’s workforce also internationally. Among study participants in this generation, workers and non-workers alike felt that Taranto’s industry was actively contributing not only to their wealth, but also to a form of Italian nation-making from which they had been previously excluded as southerners.

Taranto’s industrial pride also carried a sense of partnership with the state perceived as the enabler of social mobility and modernization. A study participant recalled that her husband’s work in the factory “gave us an identity.” Although he later died due to lung cancer and while she believed this was “unfair,” she also expressed a sense of acceptance toward the illness:

Yes, we would have made different choices had we known [about toxic exposure], but in the end his job enabled us to raise three children, have a nice house, go on holiday and do all these nice things. [...] in the end I think our family would have been very different without that job.

While this attitude toward the industry has also been described as resignation (Panico 2020) and a trade-off of employment vis-à-vis health (Barca and Leonardi 2016), our work suggests that this generation also rationalized and accepted pollution by anchoring it to processes of identity- and place-making. This generation found pride in the lives that they could live because of the factory.

Even among non-factory workers, a few participants explicitly stated that they experienced an overall improvement of quality of life thanks to industrialization. One went on to add that “pollution is better than poverty,” which highlights the importance of contextualizing the experience of toxicity within a community-specific timeframe. In addition to this, six participants perceived pollution as a collateral effect of their financial inheritance to their children. One of these mentioned: “Mario made enough money to build a summer house by the sea, now his children and grandchildren use it, too!” In the words of another participant, people in Taranto “worked in the factory so that their children wouldn’t have to work in the fields.” In this sense, although people interviewed in this generation recognized the damage caused by the industry, they also witnessed the benefits it brought about. More importantly, what these experiences illustrate is the role that subsequent generations play in framing industrialization as an investment for the future. Weaving mortality with wealth and wellbeing, these intergenerational experiences of the industry enable valuations of toxicity in which financial wellness must coexist with biological harm.

We stress that these experiences are not to be generalized to the whole community but should be rather seen as intertwining narratives that are often overlooked in Taranto’s environmental justice debate. Moving forward, our analysis shifts in the political and economic context important for understanding these different narratives from an idea of the industry as public and in the hands of a benevolent state, to a privatized entity controlled by a neoliberal economy in which residents’ wellbeing was not experienced as important (Ippolito and Walther 2019). The privatization of the factory, we argue, is connected to new understandings and narratives of industrialization and pollution, ones in which science played an essential role.

### 2nd generation (1971–2000)—‘it was our choice’: privatization and late environmental concerns

The 1980s marked a period of significant economic challenges for the European steel industry, including Taranto's steel plant. The Davignon plan, introduced to address the steel crisis, required European countries to reduce their steel production capacities (Deaglio 1983). This led to a substantial downsizing at Italsider, with over 30% of its workforce being laid off, which severely impacted the local economy. Historian Salvatore Romeo (2019) has described this moment as ‘rottura’ (breakage), a turning point marking the beginning of the industrial decline in Taranto.

Due to poor performance and mounting economic pressures, the European Union demanded the privatization of many state-owned enterprises, including Italsider. The privatization process began in 1994 and culminated in 1995 when the Riva Group acquired the steelworks and renamed it Ilva. The new management undertook significant restructuring efforts, which led to further reductions in the workforce (Pennuzzi 2001). Employment numbers dwindled from nearly 25,000 to about 12,000 as the company aimed to streamline operations and improve profitability.

The participants who witnessed this phase of industrial development without direct experience of the first phase were born between 1960 and 1995 at the time of data collection. This generation largely experienced the wealth of their parents together with the economic crisis of the 1980s, the subsequent privatization of the factory, and a sense of dissatisfaction with their economic condition, which was significantly lower than what the previous generation experienced. Their lack of satisfaction with the industry was also paired with an increasing awareness of the damaging effects of industrial activity on human health, which largely shaped the narrative of environmental injustice in the city.

When Ilva was acquired by the Riva group, things began to change. The political economic climate of the 1990s was still being affected by the global crisis of steel. The new management completely revolutionized the socio-economic conditions of the workers, first by drastically reducing the number of jobs and making them more competitive, and then by enforcing regulations to maximize efficiency on the workplace (Barca and Leonardi 2018). The episode that one ex-worker recalled encapsulates the new atmosphere in the factory:

It was a slap in the face. One day I arrived at the workplace and saw all my colleagues standing up, still like statues, looking at each other. Initially I didn’t understand, then I realized that they had taken away our chairs. All of them! Because for the new owners there was no longer room for distraction or anything like that. And so you had to spend those 8, 10, 12 hours inside the mill standing up. They also changed all our coveralls, all of them without pockets. You were not supposed to carry your phone or any other kind of distraction, you had to be there working and that was it.

With an increase of the productive capacity as the fundamental goal, the new management hit the peak of steel production in 2007. However, according to the workers interviewed in this study, this was only possible at the expenses of their quality of life, their families,’ and—more broadly—the residents of the whole city. The privatization of the factory marked another important moment in the history of the community, slowly turning the industrial dream constructed in the second half of the twentieth century into a tale of state betrayal. By maximizing production at the expense of the economic stability of the workers, the paradigm that had dominated the industrial expansion of the first decades of Ilva fell apart. As Fortun (2012) points out, this phase of late industrialism not only determines the irreversibility of the industrial decline, but also establishes the conditions for public discontent and dissatisfaction.

As the factory changed, so did the stories about it. It was precisely during these years of worsened material conditions that the idea of environmental damage and ‘the ecological question’ (Romeo 2019) entered the public discourse. Some high school teachers mobilized, producing a chemical analysis of a locally produced cheese that revealed extremely high concentrations of dioxin (PeaceLink 2008), a carcinogenic compound produced in steelmaking processes (Matés et al. 2010). These early investigations became the launchpad of epidemiological studies, advocacy initiatives and lawsuits that have made Taranto’s industrial pollution infamous worldwide. Thus, the rise of public awareness toward environmental pollution and its effects on human health can be considered as one of the byproducts of the individual discontent with the economic and occupational situation in Taranto but also in Italy more broadly. As Barca (2012, p. 73) notes in the context of Italian working-class environmentalism, a consciousness of the political link between occupational, environmental and public health was not a philosophical speculation for a few militant scientists: in fact, it was largely shared within the Left, and in the union confederations, and led to a series of social struggles both at the workshop and at the community level.

With late industrialism, what had once been the pride of thousands of families, namely the participation in a wider project of national development, started turning into a shared anxiety toward the future, a new paradigm dominated by job uncertainty and health risk (see also Barca and Leonardi 2018).

This transition to late industrialism carried also a moral conflict among residents of Taranto. One of them recalled:

Until everything went well, we did not complain. Now our jobs are also uncertain, so we start complaining, too. I think we deserved this, we should not complain. It was our choice, if we had really cared we could have foreseen the consequences, but it was convenient to bring that money home at the end of the month. So what I can do is to try and overcome my own limits, teach my child that I have made a mistake so that they will choose wisely for the future.

Much like the above quote, many of the study participants did not always frame the narrative of injustice as absolute, but instead problematized it; some of them made the point that what was later framed as harmful had once been perceived as beneficial, in one case pointing out that ‘environmentalists won’t tell you this part of the story’. The moral crisis observed in this generation manifested itself in the recognition that the Tarantini, too, played a role in this project of environmental injustice.

Moreover, as the above quote illustrates, participants in this generation seemed to be mostly concerned about their children being able to live well despite pollution, even away from the city if necessary. This approach to the next generation had its foundations in the recognition that Taranto’s problem was not simply pollution. In the neighborhoods near the factory, pollution overlapped with increased poverty and crime (see also Panico 2020). More broadly, a participant explained that the jobs that the factory once created instilled a lack of care in the residents, who became excessively finance oriented. This individualistic outlook is what participants interviewed in this generation critiqued, often illustrating this idea through the examples of the streets of the city center being littered and covered with dogs’ excrements because residents would only be interested in looking after their own private spaces (Ippolito and Walther 2019).

In other words, in this generational narrative pollution became part of a problem of which the Tarantini were also a part. Silvia, an environmental educator in her 30s, explained when talking about the future of her generation and the future she saw for the city:

For a moment we know what our problems are, but we never talk enough about our own resources and our need to change our point of view on the great work we have to do in the next ten to fifteen years. That is, we must work our asses off if we want something to change here; […] blaming the industrial pole alone is an excuse. The industrial centre will always stay there if we don't manage to build alternatives and prove to ourselves and the outside world that we don't want that thing and that we can live off something else. For now, there is nothing else.

Silvia had the privilege of not depending on a factory job to survive. However, much like others in this generation, she was dependent on part-time and insecure employment and had therefore worked away from the city for many years. In this generation, we found that some participants had relatives who had previously worked in the factory, but who—much like Silvia—did not demonstrate the same emotional attachment to the industry as their parents’ generation. A key finding here was their experience of the hardship in relation to industrial development without the benefit of its material wealth, which brought residents in this generation to craft a new narrative of Taranto’s environmental problem.

This new narrative in which industrialization and wellbeing were disconnected was reinforced by considerations made by some participants about their parents moving to Taranto for work and without any social ties to the city. To people like Silvia, this implied growing up in places where they “did not belong,” disconnecting them from the place. In our conversation, Silvia reflected on this lack of social ties to explain why it was hard for her to feel attached and care for the place:

I don't know if I will stay here, I am not particularly fond of this city. […] I mean, I have nothing here but also because my parents are not from here. I mean, I was born here by chance, because they came here for different work reasons and they stayed here. So I don't really have these roots...all this great bond here, I mean I don't really have this great bond with any place. This place needs care and so basically, I have understood that wherever you live, even if it's for a short time, you have to take care of it.

Other participants’ experiences resonated with Silvia’s. Industrialization in Taranto implied a significant economic shift in the region, moving people from neighboring cities to work in the factory or in other businesses that were growing as a result of industrialization. This economic reconfiguration of the city also reshuffled the city’s social composition, with a resulting sense of uprootedness on behalf of the second generation observed. This lack of belonging, Silvia and other participants argued, was to attribute in part to other social issues that overlapped with pollution in the city, and that were not accounted for in environmental justice accounts of industrialization (see also Jokela-Pansini and Militz 2022). At the same time, this sense of non-belonging laid the foundations for a renewed sense of belonging in the third generation, as we observe in the next section. Put together, these diverging intergenerational narratives are to be understood as active parts of a changing environment.

### 3rd generation (2001–present)—‘it's perfect despite its imperfections’: environmental lawsuits and children

The early 2000s saw a growing recognition of the severe environmental and health impacts of Ilva's operations in Taranto. In 2001, public prosecutors in Genoa began investigations into the environmental practices of Ilva, leading to the closure of its operations in Genoa between 2002 and 2005. The productive capacity of Genoa was transferred to Taranto, which intensified the environmental burden on the local community (Bonelli 2014).

Environmental and public health concerns escalated and resulted in legal actions against Ilva's management. In 2002 and 2007, criminal courts found the company guilty of environmental violations and acknowledged the excessive pollution and its detrimental effects on public health (Alliegro 2020). These legal battles fuelled public outrage and led to the rise of environmental advocacy movements, such as the Alta Marea (High Tide) movement, which campaigned vigorously between 2008 and 2012. In 2015, the Italian government placed Ilva under special administration to balance production reduction with employment protection and prepare the facility for potential acquisition (Casula 2015). ArcelorMittal's acquisition of Ilva in 2017 marked a new chapter of public–private co-ownership, but environmental issues persisted. In 2019, the European Court of Human Rights ruled that the Italian government had failed to protect Taranto’s residents from industrial pollution, leading to further legal and political conflicts (Greco 2020). Activists, increasingly frustrated with the slow pace of change, sought international intervention, appealing to bodies like the United Nations to pressure the Italian government into taking more decisive action. To infuse their claims with a sense of urgency, Taranto’s activists highlighted the severe consequences of pollution across generations.

Activism in Taranto played an important role in raising public awareness of industrial pollution and its related issues. By documenting and sharing residents' daily experiences of living with toxicity, activist groups gradually established distinct objectives and methods. Demands varied significantly, from advocating for the complete closure of the factory to promoting its ecological transition. This diversity in goals led to several ruptures among groups, complicating efforts to establish unified strategies.

A particularly contentious and unresolved issue remained the future of factory workers, who would lose their employment if the facility were shut down, especially given that viable economic alternatives to industrial production have only recently started emerging in Taranto. The resulting tension between those prioritizing job security and those emphasizing health concerns further deepened divisions within the community. Such polarization tends to fragment collective efforts and makes it challenging to pursue coordinated action. Meanwhile, younger generations appeared increasingly disengaged from the fate of the steel industry, often envisioning their futures away from the city. Taranto’s youth and its relationship to the city became increasingly central to the environmental debate and prompted new discussions around individual and generational responsibility in the face of industrial harm.

**Fig. 2:**
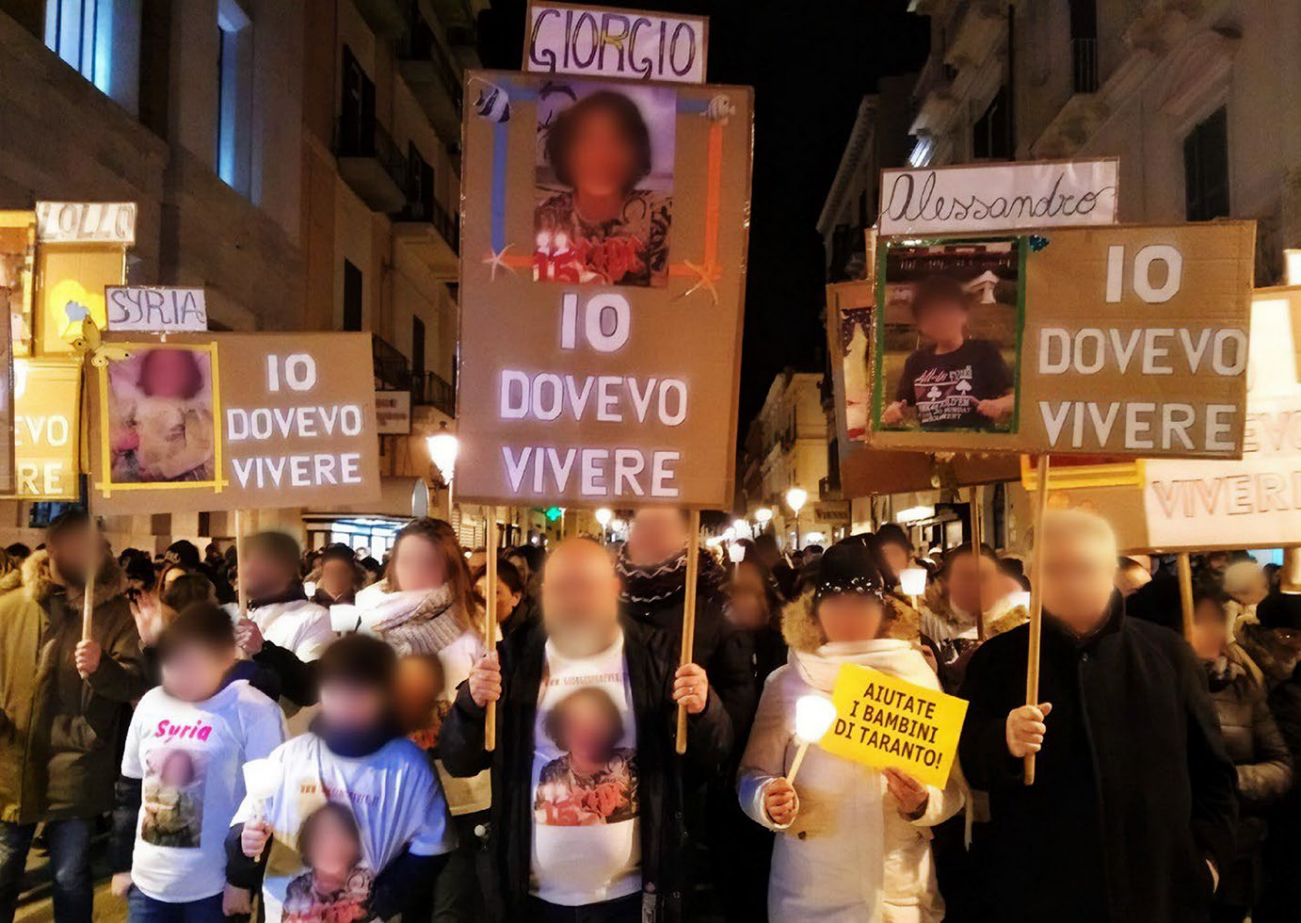
Taranto’s families march to remember children who died due to pollution-related illness. Banners read ‘Io dovevo vivere’ (I was supposed to live) (Inchiostroverde.it, 2019)

As Janelle Lamoreaux (2021) writes, “the value of the intergenerational for environmental activists and scientists is, then, that it has the potential to galvanize public perceptions of harm at scales that exceed the individual” (540). For example, more recent appeals built on studies documenting the increased mortality and neurocognitive disorders resulting from heavy metals contamination among children (Lucchini et al. 2019; Renzetti et al. 2021). A landmark trial was initiated by the family of Lorenzo Zaratta, a 5-year-old child that died due to a rare form of brain tumor. An autopsy later detected alarming concentrations of heavy metals in the brain of the child (Casula 2024). Building on this and similar cases, environmental activists interviewed in our research often described children as the ultimate victims of environmental injustice. Associations like ‘Genitori Tarantini’ (Tarantini Parents) were established out of a growing concern for children’s health. Activists often framed their claims around children to evoke a sense of urgency to act on the injustice and suffering that affected the city (see Fig. 2). While this approach to the younger generation has proven productive in advancing environmental justice demands (Powell and Stewart 2001), the voices of children and teenagers are often left out of official environmental narratives (Makuch and Aczel 2020). Here, activist narratives often unintentionally flattened the generational agency of Taranto’s youth, reinforcing their image of passive victims destined to succumb to environmental harm or fled the city.

In this part of our paper, we depart from Taranto’s activist narratives on children and instead offer an overview of their own representations of toxicity and the industry. We turn to under-age residents (6–17), to understand how environmental narratives have been impacted by the industrial development described above. This approach, we show, does not necessarily align with environmental advocacy claims, but rather sheds light on how toxicity is a multifaceted and heterogeneous experience. We recognize that this generation is inherently different from the previous two generations because not yet being of working age. This positions them differently and outside the economic dependency and pride dynamics that were central to the environmental narratives of the other two generations. Here we ask the question: how do children frame their experience of industrial toxicity in conversation with the former two generations? What are the environmental narratives emerging from this dialogue?

Through a storytelling-based exercise (Hildebrandt et al. 2016; Lewis and Hildebrandt 2020), Carmen Sale (co-author) asked 120 children in Taranto to produce fictional stories set in their neighborhood. Unlike the first and second generations who reflected with us on their lived experience, we focused on the children’s environmental narratives through a combination of both lived and imaginary narratives. With this exercise, we sought to investigate both the material and discursive elements that shape children’s experience of toxicity. This storytelling approach is sustained by studies with children in children geography and sociology, which has proven to be more appropriate compared to interview methods used with the other two age groups (Delamont 2012; Ochs and Capps 2001; Salans 2004).

Given the creative nature of this exercise, the stories produced varied greatly in terms of content, though they all shared some common narrative elements. First, this generation seemed greatly affected by the precarious socio-economic conditions of their family and their neighborhoods close to the factory, as we show below. Second, the stories also displayed an awareness of the health effects of industrial pollution from an early age, which in the other two generations developed gradually over time. Many of the stories begin this way:

[...] This friend lived in the famous steel neighborhood, which was grey and uninhabited [...] one day they found a lamp, closed their eyes and woke up in a beautiful city without the monster.

In this case, the “famous steel neighborhood” is Tamburi, the closest to the factory. As this participant explained, it is famous because “everyone knows it is polluted, people get sick and sooner or later everyone will leave.” In the story, the participant found a solution to the damage caused by pollution by making the factory disappear altogether through a magical lamp. This demonstrates the child’s strong awareness of the role that the factory plays in the decay of the neighborhood.

Participants in this generation also associated the grayness and starkness of their neighborhood to describe its deviation from ideals of urban wellbeing that rely on principles of environmental sustainability, good health, and education. The data we collected shows that, especially in neighborhoods closer to the factory, this lack of environmental wellbeing was internalized by some of the participants as stigma, a territorial affiliation with the polluted landscape, as highlighted by a 10-year-old: “we are Tamburi’s children, we are polluted inside.”

With this awareness toward the negative effects of pollutants on the community, however, also came an attention toward the positive qualities of the polluted place. The stories collected sometimes built on elements of social cohesion that emerged despite the hardship faced in the neighborhood, reinforcing a sense of emotional attachment to place:

In my neighbourhood there are a lot of good people but also not so good people... It is penalized due to the presence of the factory [...] People elsewhere discriminate against Tamburi, but to me it’s perfect despite its imperfections.

Another participant mentioned: “Even if I don't like this city, I still feel happy because I have my friends and my relatives. For this reason, it's beautiful, too.” Inherent to this sense of beauty that this and other participants described, is the idea of social connection and resilience. The children interviewed expressed pride in their community’s ability to stand and strive for normality amidst pollution. It is in this process of daily perseverance and resilience that this generation constructs a new shared identity that appeared more resigned in the other two generations (see also Clauss-Ehlers and Weist 2004 and Goldstein and Brooks 2012).

We argue that in the third group, the core of these environmental narratives is an attention to community cohesion to overcome industrial harm. In some cases, pollution in the more polluted neighborhoods seemed to be more a deep-rooted matter of social fragmentation than solely related to public health. Industrial decay, social instability, and the concentration of certain lifestyles and values in more socio-economically deprived areas of the city became the foundation for a new type of sociality. In Tamburi, this sociality is encapsulated in the idea of “l’arte di arrangiarsi,” that is, ‘the art of’ adjusting one's condition as best one can, which often shades into illegality, similarly to Valdivia’s work on ‘jugarse la vida’ in the context of an oil town in Ecuador (Valdivia 2018).

Another participant wrote in their fictional story:

A boy named Valentino lived in the Tamburi neighbourhood, and his friends used to tease him because the place where he lived is not a good place and the people who lived there are not good people. But Valentino took them around the neighbourhood one day and showed them that even though it was polluted, it was full of beautiful things […].

Similarly, another story narrated:

C. and S. are two inseparable friends... One day, tired of being oppressed by the dust, they ask their parents to leave. Their parents, however, teach them that running away is wrong; on the contrary, one must fight together.

These narratives highlight how some of this generation’s participants perceive their agency as rooted in community solidarity, serving as a shielding factor against the adverse effects of pollution. The stories emphasize themes of friendship, overcoming adversity, positive change, respect for their neighborhood, and the importance of not judging others based on prejudice. Collective efforts to reinterpret the narrative of toxicity create a supportive setting for both children and the broader community (Jencks and Mayer, 1990).

## Intergenerational narratives of toxicity and the pursuit of a ‘good life’

This study has examined how experiences of pollution in Taranto unfold across three generations, each shaped by shifting material conditions and evolving understandings of harm. Among the first generation, the steel factory provided economic relief and a sense of modern identity that lifted families out of poverty. This industrial promise solidified both personal pride and faith in the state’s developmental agenda. The advent of the factory saw Taranto’s transformation from a predominantly agricultural society to an industrial power with a new middle class. Participating in this project of industrial development also brought forth a desire to participate in a modern Italian society, a project from which the South had been systemically excluded since the unification of Italy (Gramsci et al. 1994). This process of industrial, urban and social transformation also redefined what constituted a ‘good life’. To the first generation, this meant actively contributing to the project of Italian modernity, striving toward personal and national economic growth. For workers of this generation, care was enacted through materially providing for their families.

In contrast, the second generation, shaped by emerging scientific evidence of pollution’s health impacts, challenged their parents’ interpretation of wealth. For them, the initial narrative of economic prosperity came to be seen as a precarious fiction, as revelations about toxicity reframed the landscape in terms of injustice and disillusionment. In this generation, the idea of ‘good life’ shifted, highlighting the dilemma of the juxtaposition of good health vis-à-vis employment. As late industrialism (Fortun 2012) took hold, it reshaped not only economic conditions but also the very experience of space. By the late 2000s, places once rich with shared meaning and collective practice began to erode into spaces marked by struggle and fragmentation, revealing a broader weakening of the collective fabric that had once supported community life. Based on this new awareness, ideas of care also began to shift from materially sustaining the family to taking action over environmental health concerns.

The third generation, growing up amid enduring contamination and economic instability, developed narratives of resilience and cohesion and rooted their identity in the everyday struggle to persist and find beauty in a damaged environment. As one participant emphasized, “we need to change what is wrong in order to grow together, no one should be forced to leave.” Despite depopulation and mounting pressures, this generation gathers around not only shared suffering but also a sense of dignity rooted in collective history. This generation was no longer occupationally tied to the industry due to the decay of industrial activity. To this generation, a ‘good life’ is defined by the ability to reclaim collective agency in the face of industrial harm, asserting their belonging to the city and their neighborhoods despite the painful awareness of the irreversible industrial damage. Here, care emerges as a commitment to the previous generations to take action on the injustice that the city suffered without complete awareness of the community. Very different, yet connected to the environmental subjectivities of the previous two generations, the experience of this generation shows how intergenerational narratives are continuously inherited, renegotiated, and enacted.

Beyond shifting perceptions of what constitutes a ‘good life,’ our analysis also stresses that environmental narratives do not merely oscillate between acceptance and rejection of industrial development, but rather are continually shaped by complex interplays of social, political and material forces in different historical contexts. For the first generation, industrial revival offered a means to strengthen social identity; for the second, such identification gave way to a sense of betrayal and the collapse of communal engagement; and for the third, renewed practices of care emerged in the face of ongoing harm. Taken together, these generational experiences highlight that identity is not fixed. Instead, as Hague and Jenkins (2004) remind us, “it is this process of receiving, reconstructing, and then re-communicating a narrative that constitutes our identity.” In Taranto, these processes of identity-making depend on the interlocking temporalities of past, present, and future, shaped by continuous human and non-human encounters, and by the evolving meaning of environmental contamination.

Building on the ways each generation reshapes environmental narratives, we stress how the youngest generation steps into a landscape already layered with the narrative legacies of their elders. They are called not only to uphold the present but also to carry forward aspirations for future redemption. In drawing on this collective past, placing emphasis on cohesion and resilience reflects a deliberate choice that highlights intergenerational dialogue as a source of shared strength rather than division.

At the same time, as these narratives unfold within shifting spatial and temporal contexts, it becomes clear that the same physical environment can yield contrasting experiences and political visions. Thus, understanding intergenerational narratives entails not only recognizing their connective tissue but also acknowledging the subtle differences that emerge when stories and values intersect, overlap, or diverge. It is in this continuous process of engagement, othering, and care that these narratives acquire their full meaning.

The idea of ‘intergenerational narratives’ to study toxicity has a two-folded implication: (1) it implies a need to shift attention to the micro-scale of environmental experience and (2) it requires to be carried across generations to understand how collective environmental subjectivities evolve over periods of time beyond individual lives.

Our analysis focused on the epistemic exchange and narratives that emerge when ‘slow observation’ (Davies 2019)—a sustained, attentive engagement with the subtle, often overlooked details of daily toxic exposure—is produced and reproduced across generations. Understanding how environmental subjectivities produce specific narratives around toxicity requires a double endeavor. One is to attune to the everyday, diluted, and intimate experience of toxicants, bringing together experiences that often stay unspoken (ibid.). The other is to simultaneously stretch the intimacy of toxicants across the temporal fabric to understand how environmental narratives are reproduced and changed over time. Our approach emphasizes how toxicity’s slow unfolding transforms from individual experiences into narratives of shared significance of a ‘good life,’ and how these narratives are constantly reinvented as they pass from one generation to the next.

## Conclusion

This paper has sought to reframe pollution and toxicity in Taranto, not as isolated events or strictly individualized struggles, but as relational, intergenerational experiences woven into the city’s social fabric and industrial landscape. A joint analysis of our data across the three different generations illustrates that narratives of pollution should be understood from a relational perspective. This includes not only how different generations understand environmental issues in relation to one another, but also how they are part of the historical and environmental landscape in which they observe change.

Within this framework in which the human and the industrial are connected, we sought to highlight how different temporal positionalities co-produce heterogeneous environmental subjectivities in the community of Taranto. Our paper expands the debate on environmental injustice in the context of industrial contamination by shedding light on the ambivalent nature of resigned and intimate responses to pollution across generations. Our joined analysis demonstrates that residents of industrial towns mitigate toxic harm with other dimensions of life. Like Lora-Wainwright (2021) and Tironi (2018), we recognize the everyday strategies that residents of Taranto adopt to resist, curb, or ethically reject pollution, but in doing so we also shift our attention to the ways in which industry and pollution enable the construction of heterogeneous narratives of toxicity across generations.

Beyond the academic exercise that we offer with this paper, we also like to think that a change of perspective on communities that are seemingly divided in the face of environmental disaster can contribute to pluralizing conversations around justice. It can be interpreted as a rich resource that fuels a dynamic process of reflection and adaptation. In recognizing the dialogical and temporal complexity of these environmental subjectivities, we do not essentialize Taranto’s experiences and narratives of pollution across generations. Instead, these often-ambivalent attachments that emerge within polluted landscapes unfold as an ongoing project of intergenerational care—one that reconnects rather than divides. Environmental consciousness, then, emerges through intergenerational dialogue, as old narratives are unsettled, contested, and transformed by new voices and experiences.

Our study thus shows that in so-called ‘left behind places’ (Pike et al. 2024) like Taranto, the categories of “contaminating” and “contaminated” are not fixed opposites but mutually constitutive forces that shape one another over decades, reflecting the city’s changing economic regimes and environmental conditions. Navigating changing economic, environmental and medical scenarios in the city also implies shifting definitions of a ‘good life’.

More importantly, moved by different definitions of ‘good life,’ each generation acts and displays forms of collective care toward the other generations. The stories shared by older residents who remember state-led development, by the middle generation disillusioned with privatization and state neglect, and by children discovering both harm and beauty in a tarnished landscape all contribute to a fuller understanding of environmental subjectivities. Each narrative strand challenges simplistic distinctions between victims and perpetrators, insiders and outsiders, and the notion of a singular environmental truth.

It is important to acknowledge that our methodological approach emerged from three separate studies, each shaped by distinct research questions, study participants, and methods. Future research might develop more integrated approaches to better capture the continuity and contrast in intergenerational narratives. Such studies may offer insights for policy and community engagement. Studying how each generation relates to pollutants differently could lead to environmental interventions and identifying more inclusive policies that resonate with communities’ historical contexts and needs.

In sum, our study brings forth the notion of intergenerational narratives of toxicity as a conceptual tool to study attachment and detachment, care and neglect, and the notion of good life situated in different historical contexts in Taranto and beyond. Through these narratives, Taranto emerges as “a resisting community created in the very experience of contamination” (Armiero et al. 2019).

## Data Availability

Neither of the three ethics protocols included in this paper allow the data collected to be made publicly available.
